# Preclinical Evaluation of Oncolytic Δ**γ**_1_34.5 Herpes Simplex Virus Expressing Interleukin-12 for Therapy of Breast Cancer Brain Metastases

**DOI:** 10.1155/2012/628697

**Published:** 2012-12-31

**Authors:** James J. Cody, Pietro Scaturro, Alan B. Cantor, G. Yancey Gillespie, Jacqueline N. Parker, James M. Markert

**Affiliations:** ^1^Division of Infectious Diseases, Department of Pediatrics, The University of Alabama at Birmingham, 1720 2nd Avenue South, Birmingham, AL 35294, USA; ^2^Division of Neurosurgery, Department of Surgery, The University of Alabama at Birmingham, 1720 2nd Avenue South, Birmingham, AL 35294, USA; ^3^Division of Preventive Medicine, Department of Medicine, The University of Alabama at Birmingham, 1720 2nd Avenue South, Birmingham, AL 35294, USA; ^4^Department of Microbiology, The University of Alabama at Birmingham, 1720 2nd Avenue South, Birmingham, AL 35294, USA; ^5^Department of Cell Biology, The University of Alabama at Birmingham, 1720 2nd Avenue South, Birmingham, AL 35294, USA; ^6^Department of Physiology and Biophysics, The University of Alabama at Birmingham, 1720 2nd Avenue South, Birmingham, AL 35294, USA

## Abstract

The metastasis of breast cancer to the brain and central nervous system (CNS) is a problem of increasing importance. As improving treatments continue to extend patient survival, the incidence of CNS metastases from breast cancer is on the rise. New treatments are needed, as current treatments are limited by deleterious side effects and are generally palliative. We have previously described an oncolytic herpes simplex virus (HSV), designated M002, which lacks both copies of the **γ**_1_34.5 neurovirulence gene and carries a murine interleukin 12 (IL-12) expression cassette, and have validated its antitumor efficacy in a variety of preclinical models of primary brain tumors. However, M002 has not been yet evaluated for use against metastatic brain tumors. Here, we demonstrate the following: both human breast cancer and murine mammary carcinoma cells support viral replication and IL-12 expression from M002; M002 replicates in and destroys breast cancer cells from a variety of histological subtypes, including “triple-negative” and HER2 overexpressing; M002 improves survival in an immunocompetent model more effectively than does a non-cytokine control virus. Thus, we conclude from this proof-of-principle study that a **γ**_1_34.5-deleted IL-12 expressing oncolytic HSV may be a potential new therapy for breast cancer brain metastases.

## 1. Introduction

Breast cancer, the most prevalent cancer among women, afflicts approximately one in eight over a lifetime. Current treatment strategies may yield cures, if applied early, but are less effective against metastatic disease, which accounts for most deaths. Between 15% and 20% of breast cancer patients are eventually diagnosed with intracranial metastases, with autopsy data suggesting that 30% or more may actually bear these lesions [1, 2]. As treatments improve for noncranial metastases, the frequency of brain metastases is increasing. For example, much therapeutic success has been achieved with the agent Herceptin, the monoclonal antibody targeting human epidermal growth factor receptor 2 (HER2) [3, 4]. However, Herceptin and commonly used chemotherapeutics do not cross the blood-brain barrier [5, 6] at therapeutically effective levels, thus maintaining the brain as a “sanctuary site” in which tumor cells are inaccessible to systemically administered therapies [6]. As a result, patients with controlled systemic disease are increasingly succumbing to the neurological complications of central nervous system (CNS) metastases. The established treatments for metastases in the brain are generally palliative [7, 8], and overall survival of patients following this diagnosis is rarely more than one year [2]. Surgery can be efficacious against solitary lesions, but patients typically present with multiple lesions that can make this approach impractical, and tumor recurrence is frequent. Radiotherapy and radiosurgery therapies have also been used for the treatment of brain metastases with modest success, but both of these approaches have limitations, including the possibility of undesirable side effects [9] that accentuate the neurotoxicities of “chemo-brain” [10] as well as an inability to treat effectively patients that have more widespread intracranial disease. It is clear that new innovative treatments are needed to combat intracranial metastases of breast cancer.

Immunotherapy is a relatively new approach to breast cancer treatment. Though breast cancers express a variety of potential tumor antigens, a given tumor may express only one of these or none at all. Furthermore, because of their heterogeneous nature, a tumor may escape immunotherapy based on a single antigen. Thus, it is unlikely that vaccines directed at single peptides will be universally successful. In contrast, cytokine-based immunotherapy has the potential to stimulate patient tumor-specific immune responses. Immunotherapy approaches have been attempted in patients with brain tumors but have thus far been limited by ineffectual systemic immunization, use of allogeneic antigens, or the inability of sufficient numbers of activated effector cells to penetrate inherent defense mechanisms of the CNS.

 Another novel anticancer strategy is the use of oncolytic virotherapy. Oncolytic viruses, including those that are naturally occurring (e.g., reovirus) and those that can be readily genetically engineered (herpes simplex virus, adenovirus), are viruses that are able to infect, replicate in, and lyse tumor cells, with subsequent spread to neighboring cells [20]. In theory, oncolytic viruses have the potential to eliminate entire tumors. Preclinical studies have demonstrated that oncolytic viruses can infect and lyse a variety of malignancies, including breast cancer, and their safety has been shown in clinical trials. At least three different oncolytic viruses have demonstrated the potential for efficacy in models of breast cancer brain metastasis, including reovirus [21], poliovirus [22], and G207, a genetically modified, conditionally replication-competent herpes simplex virus (HSV) [23], establishing the feasibility of this approach. However, antitumor efficacy of an oncolytic virus depends in part upon the extent of tumor cell transduction following the initial administration. Tumors are composed of both malignant and nonmalignant cells and include extracellular matrix barriers that can limit intratumoral spread and ultimately the effectiveness of these agents [24]. 

 Given this background, we propose a strategy that combines both of the above approaches. In this scenario, the oncolytic effect of viral replication enhances immunotherapy by reducing tumor burden and releasing potential tumor antigens; likewise, immunotherapy enhances virotherapy by the activation of an immune response against uninfected tumor cells. We have previously described a cytokine-expressing HSV, which contains deletions of both copies of the HSV neurovirulence gene *γ*_1_34.5 [25]. This virus, designated M002, does not replicate in normal cells but efficiently kills tumor cells. M002 HSV also carries an inserted gene for murine interleukin-12 (IL-12), a proinflammatory cytokine with additional antiangiogenic properties [26, 27]. Production of IL-12 thus extends the antitumor activity of M002 to uninfected cells. We have previously shown that M002 can induce an antitumor immune response and significantly enhance survival in murine models of primary brain tumors [25, 28, 29]. However, this virus has not been previously evaluated as a therapy for breast cancer. Our presumption is that the antitumor activity of M002 is not exclusive to primary brain tumors, and that it will be effective against tumors originating from other tissues, including breast cancer. This presumption is supported by both the observation that other described oncolytic viruses have demonstrated activity against multiple tumor types, and by the results of our preliminary experiments utilizing multiple tumor cell lines. 

 To test the hypothesis that this IL-12-expressing oncolytic HSV might be suitable for use against breast cancer lesions metastatic to the CNS, we assessed both human breast cancer and murine mammary carcinoma cell lines for levels of viral IL-12 production, susceptibility to viral infection and oncolysis, and viral spread over time. Additionally, an immunocompetent *in vivo* model was developed, and therapeutic efficacy in this model was examined. The results of these experiments are as follows: (i) both human breast cancer and murine mammary carcinoma cells efficiently produce IL-12 when infected with M002; (ii) M002 replicates robustly in a panel of breast cancer cells representative of brain metastases and mediates a directly cytotoxic effect in both human and murine cells; (iii) the intracranial injection of SCK cells can be used as an appropriate and reliable immunocompetent model for breast cancer brain metastases; (iv) M002 extends the survival of treated mice more effectively than does a non-cytokine control virus or mock treatment. Collectively, these results suggest that an IL-12-expressing *γ*_1_34.5-deleted HSV may be an effective therapy against breast cancer brain metastases and provide the rationale for further testing.

## 2. Materials and Methods

### 2.1. Cells

Vero cells were obtained from the American Type Culture Collection (ATCC, Rockville, MD, USA) and were cultured in Minimal Essential Medium (MEM; Cellgro, Mediatech Inc., Herndon, VA, USA) containing 7% heat-inactivated fetal bovine serum (FBS; Hyclone, Logan, UT, USA). The 4T1 murine mammary carcinoma cell line (derived from BALB/c mice) was purchased from the ATCC. The SCK murine cell line was isolated from a spontaneous mammary carcinoma in a female A/J mouse and was provided by Dr. C. W. Song (University of Minnesota Medical School, Minneapolis, MN, USA) [30]. Both lines were maintained in RPMI containing 10% FBS. The human breast cancer cell lines MDA-MB-361, MDA-MB-231, ZR-75-1, SK-BR-3, and BT-474 were obtained from ATCC and maintained in a 50 : 50 mixture of Dulbecco's modified Eagle medium and Ham's nutrient mixture F-12 (DMEM/F12) supplemented with 2.6 mM L-glutamine and 10% FBS. BT-474 cells are derived from a solid invasive ductal carcinoma; they are epithelial in morphology and establish nodules in nude mice [11]. MDA-MB-231 is an adenocarcinoma cell line obtained from a pleural effusion [15, 31]. MDA-MB-361 are adenocarcinoma cells derived from a brain metastasis [15, 31] that establish slow-growing intracranial tumors in mice when delivered by intracarotid injection [32]. SK-BR-3 is an adenocarcinoma cell line [18]. ZR-75-1 are derived from an ascitic effusion, from a patient with invasive ductal carcinoma [19]. All cells were maintained in Corning tissue culture plasticware.

### 2.2. Viruses

The HSV-1 (F) strain, which was used as a prototype for all mutant HSVs in these studies, is a low passage clinical isolate that has been previously described [33, 34]. R3659 is a *γ*_1_34.5-deleted virus that has also been described previously and was used as a control [35]. M002 is a *γ*_1_34.5-deleted oncolytic HSV that carries two copies of a murine IL-12 (mIL-12) expression cassette under the transcriptional control of the murine early growth response-1 (EGR-1) promoter and has been described previously [25].

### 2.3. Cytokine Expression

4T1, SCK, and MDA-MB-361 cells were seeded in 6-well plates at 4 × 10^5^ cells/well and then infected with M002 at a multiplicity of infection (MOI) of 1 plaque-forming unit (PFU) per cell. After 24, 48, and 72 h, supernates were harvested. The concentration of IL-12 in the samples was then assessed using an enzyme-linked immunosorbent assay (ELISA) kit specific for the p70 subunit of murine IL-12 (R & D Systems, Minneapolis, MN, USA). Plates were read with an EL_x_ 808 Ultra Microplate Reader (Bio-Tek Instruments Inc., Winooski, VT, USA).

### 2.4. Viral Replication

Both single-step and multistep viral replication assays were performed as previously described [36]. To assess viral replication efficiency in a single-step replication assay, a panel of breast cancer cells (MDA-MB-361, MDA-MB-231, ZR-75-1, BT-474, and SK-BR-3) were seeded in 6-well plates and then infected with HSV-1 (F), R3659, or M002 at an MOI of 5 PFU per cell. After 48 hours, bright-field photomicrographs (100x magnification) were taken, and the cells were then harvested and subjected to three rounds of freeze/thawing followed by sonication. Supernates were then serially diluted and titered on monolayers of Vero cells. To assess the efficiency of viral replication and spread over time, monolayers of Vero, MDA-MB-361, SCK, or 4T1 cells were infected with HSV-1 (F), M002, or R3659 at an MOI of 0.1 PFU/cell. Cells were harvested at regular intervals (12, 24, 48, 72, and 96 h post-infection), and viral progeny were titered as above.

### 2.5. Oncolytic Potency

The oncolytic potency of M002 on breast cancer cells was assessed as follows. Human MDA-MB-361 breast cancer or murine SCK and 4T1 mammary carcinoma cells were seeded in 96-well plates and then infected with HSV-1 (F) or M002 at MOIs of 1 and 10 PFU/cell. At regular intervals post-infection (24, 48, 72, and 96 h), cell viability was assessed by 3-[4,5-dimethylthiazol-2-yl]-2,5-diphenyl tetrazolium bromide (MTT) assay (Sigma, St. Louis, MO, USA), in accordance with the manufacturer's specifications. The plates were read with an EL_x_ 808 Ultra microplate reader (Bio-Tek Instruments Inc.) at 570 nm (690 nm background).

### 2.6. Tumor Survival Studies

All animal experimental protocols were carefully reviewed and approved by the University of Alabama at Birmingham (UAB) Institutional Animal Care and Use Committee, which has maintained accreditation with the Association for Assessment and Accreditation of Laboratory Animal Care International (AAALAC) since 1971. UAB is a United States Department of Agriculture-(USDA-) licensed animal research facility and an Animal Welfare Assurance is on file with the Office of Laboratory Animal Welfare. Thus, all protocols were in accordance with USDA Animal Welfare Regulations, as well as the Public Health Service Policy on the Humane Care and Use of Laboratory Animals and the National Research Council Guide for the Care and Use of Laboratory Animals.

The intracranial injection of tumor cells has been detailed previously [37] and is briefly summarized here. Female A/J mice, 4–6 weeks old, were obtained from the National Cancer Institute-Frederick (Frederick, MD, USA) and allowed at least one week for acclimation prior to beginning the experiments. For the establishment of intracranial tumors, SCK cells were harvested with trypsin, washed, and then resuspended in 5% methylcellulose in MEM at two concentrations: 2 × 10^6^ and 1 × 10^7^ cells/mL. The cells were loaded into a 250 *μ*L Hamilton syringe and kept on ice. The mice were weighed, anesthetized with an intraperitoneal (IP) injection of a ketamine (10 mg/100 g)/xylazine (1.5 mg/100 g) mixture, and the surgical site was prepared. Eight mice in two cohorts (*n* = 4, each) were then stereotactically injected in the right caudate nucleus [36, 38] with 1 × 10^4^ or 5 × 10^4^ cells/5 *μ*L, respectively, and the mice were monitored for survival. By days six (5 × 10^4^ cohort) and nine (1 × 10^4^) post-injection, all mice in each cohort had been euthanized due to the onset of neurological symptoms, in accordance with institutional guidelines. The brains were fixed in formalin and paraffin embedded, and sections of tissue 10 *μ*m thick were stained with hematoxylin and eosin for histological analysis. 

For treatment experiments, SCK cells were prepared at 5 × 10^5^ cells/mL, and then 2.5 × 10^3^ in 5 *μ*L were stereotactically injected as above. Four days later, M002 or R3659 (*n* = 10 each; 1.5 × 10^7^ PFU in 10 *μ*L) or vehicle only (*n* = 9; sterile saline) was injected at the same coordinates (intratumoral), and the mice were monitored for survival. In all survival experiments, a log-rank comparison of Kaplan-Meier plots calculated for each group was conducted on a pairwise basis.

## 3. Results

### 3.1. Breast Cancer Cells Infected with M002 Produce IL-12

**Figure 1:**
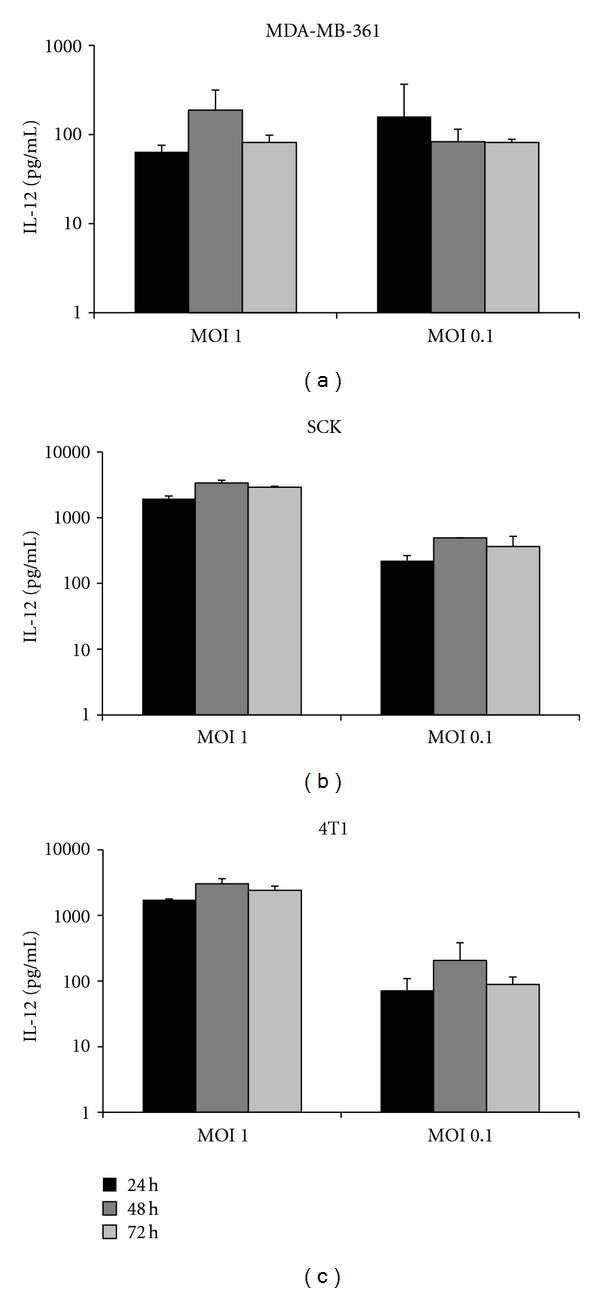
IL-12 production from breast cancer cells infected with M002. 4 × 10^5^ human breast cancer cells (MDA-MB-361) or murine mammary carcinoma cells (SCK, 4T1) were infected with M002 at an MOI of 1 or 0.1 PFU/cell. At 24, 48, and 72 h post-infection, media were harvested and subjected to an ELISA specific for the p70 subunit of murine IL-12. No IL-12 was detected from uninfected cells.

Prior to beginning *in vivo *studies, verification of IL-12 transgene expression at physiologically relevant levels from breast cancer cells infected with M002 was necessary. To this end, the breast cancer cell line MDA-MB-361, originally isolated from a brain metastasis, was selected as a representative human line for study. In addition, two murine mammary carcinoma cell lines (SCK and 4T1) were also assayed, as these cells are syngeneic with two of the immunocompetent murine models used in our laboratory (A/J and BALB/c, resp.,). The cells were seeded in 6-well tissue culture plates and then infected with M002 at a multiplicity of infection (MOI) of both 0.1 and 1 plaque-forming units (PFU) per cell. At regular intervals post-infection (24 h, 48 h, and 72 h) cultured medium samples were harvested and then subjected to an enzyme-linked immunosorbent assay (ELISA) specific for the p70 subunit of murine IL-12. For all three lines, little to no endogenous IL-12 production was detected from uninfected cells. In contrast, infected cells yielded a high level of IL-12 production across all three time points, with a slight trend towards maximum production at 48 h (Figure 1). Amounts of IL-12 ranged from approximately 100 pg/mL to greater than 1000 pg/mL, with higher levels obtained from the murine lines. For both SCK and 4T1 cells, infection at an MOI of 1 yielded approximately 10-fold more IL-12 than infection at an MOI of 0.1. For the MDA-MB-361 cells, the level of IL-12 expression was similar at both MOIs tested (1, 0.1). Overall, these data confirm that both human breast cancer and murine mammary carcinoma cells support IL-12 production following infection with M002.

### 3.2. M002 Replicates in Both Human Breast Cancer Cells and Murine Mammary Carcinoma Cells

**Figure 2:**
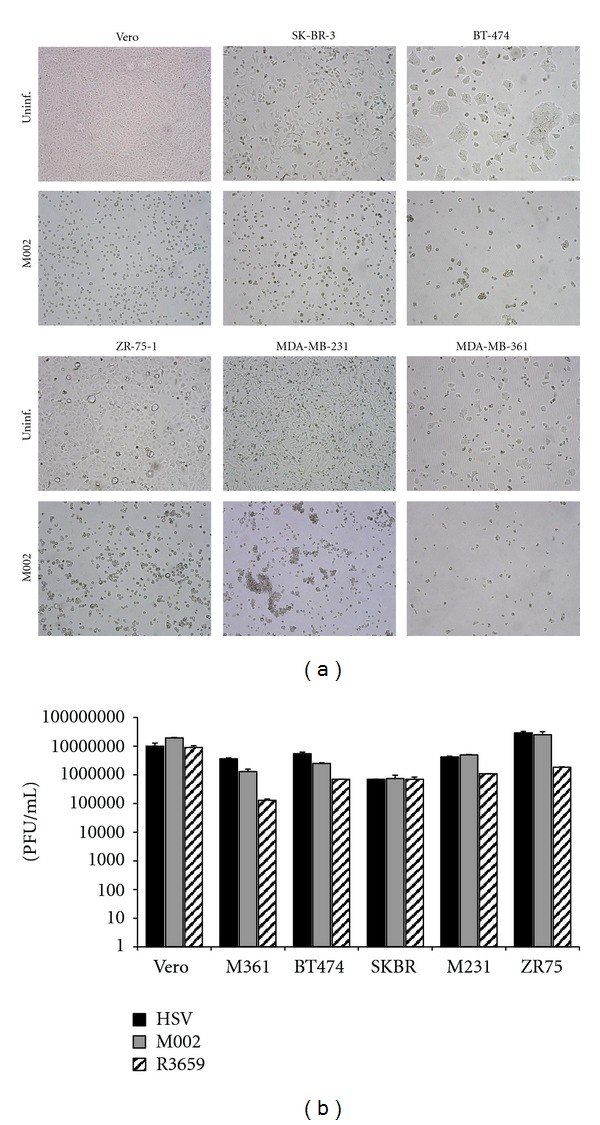
A panel of cell lines including permissive Vero cells and 5 human breast cancer cell lines, MDA-MB-361 (M361), BT-474, SK-BR-3 (SKBR), MDA-MB-231 (M231), and ZR-75-1 (ZR75) were infected with M002 at an MOI of 5 PFU/cell. (a) Bright-field photomicrographs (100x magnification) of viral cytopathic effect mediated by M002 in the infected cells shown in comparison with healthy uninfected cells. The cytopathic effect mediated by HSV-1 (F) and R3659 (not shown) were identical to M002. (b) Replication of wild-type HSV-1 (F), IL-12-expressing HSV M002, or the non-cytokine-expressing control virus R3659 in a panel of human breast cancer cells at 48 h post-infection. Shown are the averages and standard deviations of duplicate determinations, from a representative of three experiments.

A number of studies on breast cancer patients have determined that the following factors are associated with increased risk for brain metastasis (reviewed here [39, 40]): younger age, premenopausal status, invasive ductal carcinoma (IDC) histological type, estrogen receptor negative (ER^−^), p53 mutations, and EGFR overexpression. Additionally, there are strong associations with both HER2 overexpressing and “triple negative” (ER^−^, progesterone receptor-negative, HER2^−^) tumor types and brain metastasis [41, 42]. To evaluate whether M002 displayed oncolytic activity against breast cancer brain metastases, viral replication was assessed *in vitro *in a panel of human breast cancer cell lines representative of some of the aforementioned characteristics (Table 1). In a single-step replication assay, cells were plated as above and then infected with M002 at an MOI of 5 PFU per cell. At 48 h post-infection, photomicrographs were taken, and cells and media were then harvested and subjected to three rounds of freeze/thawing and sonication. Serial dilutions of the lysates were then titered on monolayers of the permissive Vero cell line. As shown in Figure 2, all cell lines displayed marked cytopathic effect and yielded robust progeny virus production. In most lines, the replication of M002 was similar to that of wild-type HSV-1 (F) and was within 1-2 orders of magnitude of the levels reached in the control Vero cells. This result confirms that *γ*_1_34.5-deleted HSVs such as M002 are able to replicate efficiently within these breast cancer cell lines. 

**Figure 3:**
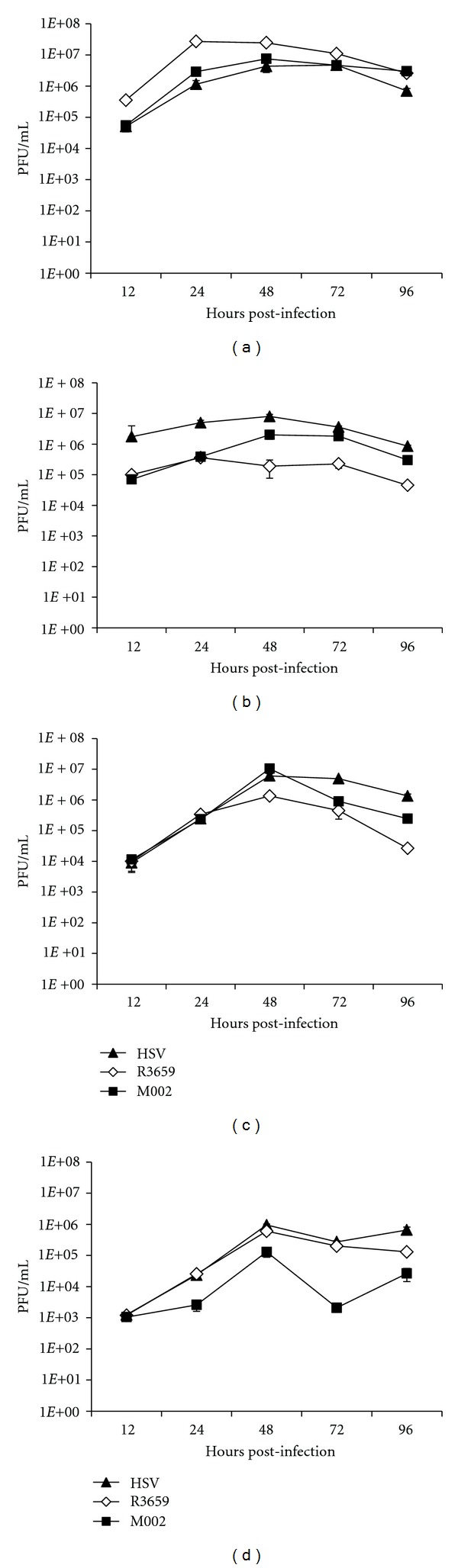
Replication of M002 over time. In a multistep replication assay, HSV replication was assayed in the following lines: the permissive Vero cell line (a), human breast cancer MDA-MB-361 (b), and murine mammary carcinoma lines SCK (c) and 4T1 (d). Cells were infected with HSV-1 (F) (triangles), R3659 (diamonds), and M002 (boxes) at an MOI of 0.1 PFU/cell. At multiple time points, cells and media were harvested, and viral replication was assayed by titration on Vero cell monolayers.

 To further analyze viral replication, a multistep replication assay was employed to assess the efficiency of viral spread. In this experiment, replication in MDA-MB-361 cells was compared to that in SCK and 4T1, as well as Vero cells as a control. Cells were infected at an MOI of 0.1, and then progeny virions were titered as above at 12, 24, 48, 72, and 96 h post-infection. Overall, robust replication was observed in all cell lines, with the progeny titers increasing rapidly from 12 to 48 h and then declining somewhat by 96 h (Figure 3). Maximum titers were reached at 48 h post-infection in all cell lines. It was observed that viral replication in the MDA-MB-361 cells was within one log of that seen in the Vero cells, indicating that these cells are also highly permissive for HSV replication. A similar level of replication was seen in the SCK cells. In contrast, the 4T1 cells were more resistant to replication, with the maximum titer of progeny M002 less than the other lines by more than one log at 48 h post-infection. This relative resistance of the 4T1 line to HSV replication has been reported by others [43].

### 3.3. M002 Exhibits Oncolytic Potency in Breast Cancer Cells

**Figure 4:**
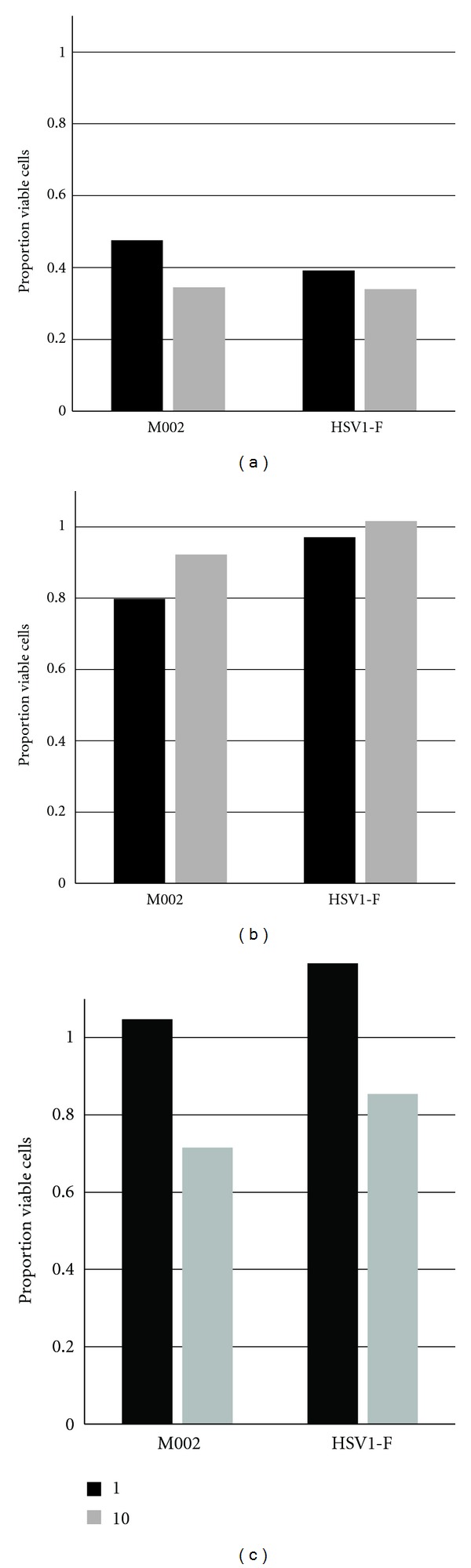
Cytotoxicity of M002 in human and murine tumor cells at 48 h post-infection. Plates of human breast cancer cells MDA-MB-361 (a) or murine mammary carcinoma cells SCK (b), and 4T1 (c) were infected with the indicated viruses at an MOI of 1 (black bars) or 10 (gray bars) PFU/cell. At 48 h post-infection, cell viability was assayed by MTT assay. Shown are the averages of quadruplicate determinations, normalized to uninfected cells and expressed as the proportion viable cells.

The oncolytic potential of M002 in breast cancer cells was assessed as follows. Both human (MDA-MB-361) and murine (SCK, 4T1) cancer cells or Vero control cells were seeded in multiwell plates and then infected with wild-type HSV-1 (F) or with M002 using MOIs of 1 and 10. Cell viability was assessed by MTT assay at multiple time points. In the control permissive cells (Vero; not shown), infection with M002 or HSV-1 (F) wild-type strain reduced cell viability to approximately 30% relative to uninfected cells by 24 h, and that effect persisted through 96 h. In the MDA-MB-361 cells, viability was less affected at 24 h, but for both viruses, cell viability was reduced by greater than 50% at 48 h (Figure 4) and declined further by 96 h to approximately 20%. In contrast, the SCK cells exhibited little reduction in viability by 48 h, although viability declined to less than 20% in M002-infected wells by 96 h. In accordance with our replication data, the 4T1 cells were less susceptible to HSV-mediated cytotoxicity. Viability was reduced only approximately 30% (MOI = 10) by either virus at 48–72 h and did not decrease further by 96 h. Overall, these results mirrored those of the replication experiments, and indicate that infection with M002 is directly cytotoxic to breast cancer cells. 

### 3.4. Treatment with M002 Prolongs Survival in a Novel Model of Breast Cancer Brain Metastases

**Figure 5:**
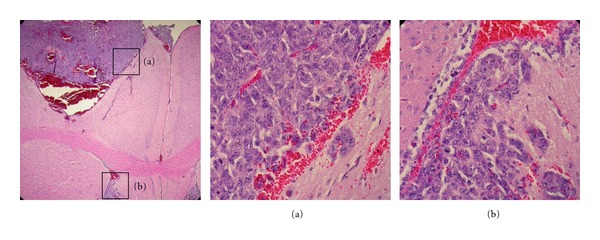
Development of an immunocompetent intracranial model of breast cancer brain metastases. Female A/J mice in two cohorts (*n* = 4, each) were given intracranial injections of 1 × 10^4^ or 5 × 10^4^ cells/5 *μ*L. By days six (5 × 10^4^ cohort) and nine (1 × 10^4^) post-injection, all mice in each cohort had been euthanized due to the onset of neurological symptoms. Brains were harvested for histological examination. Shown are hematoxylin and eosin-stained sections from a representative specimen. Note the massive tumor at the injection site in the first panel (40x magnification) with associated hemorrhaging. Insets (400x) show the relatively well circumscribed border of the main tumor (a) and the more invasive character of a distant tumor nest (b).

Having established that breast cancer cells support IL-12 production from M002, viral replication, and are susceptible to M002-mediated cell death, we next evaluated the potential for M002 as a therapy for breast cancer brain metastases in a preclinical model. M002 has demonstrated efficacy against primary brain tumors in a number of preclinical models [25, 28, 29, 44, 45], but it has not yet been tested against metastatic brain tumors. Existing models of breast cancer brain metastasis typically involve the use of genetically modified or *in vivo* passaged human cell lines inoculated into immunodeficient mice [46, 47]. Because M002 expresses murine IL-12, a maximum antitumor effect is achieved only in the context of an intact immune system. For this reason, we decided to examine the effectiveness of M002 in an immunocompetent murine model. The A/J mouse strain has been previously used by us to evaluate M002 in models of neuroblastoma [25, 29, 44] and is particularly useful from a safety standpoint due to the sensitivity of A/J mice to wild-type HSV replication. Moreover, the SCK cells were more similar to the human cell lines in terms of permissiveness for M002 replication and cell death. To model brain metastases, SCK cells were injected into the brains of mice at two different doses (1 × 10^4^, *n* = 4; 5 × 10^4^, *n* = 4), and the mice were monitored for survival. We observed rapid intracranial tumor growth *in vivo*, requiring the euthanization of the mice given the higher dose by 6 days post-injection and those given the lower dose by 9 days post-injection. Brains of the mice were harvested and examined histologically. As shown in Figure 5, the tumors were relatively well circumscribed in general. The establishment of distant tumor nests, particularly in the ventricles, was frequently noted. These nests tended to be somewhat more invasive into the brain parenchyma than were the main tumors. These tumors were associated with hemorrhages, suggesting “leakiness” of the tumor vasculature.

**Figure 6:**
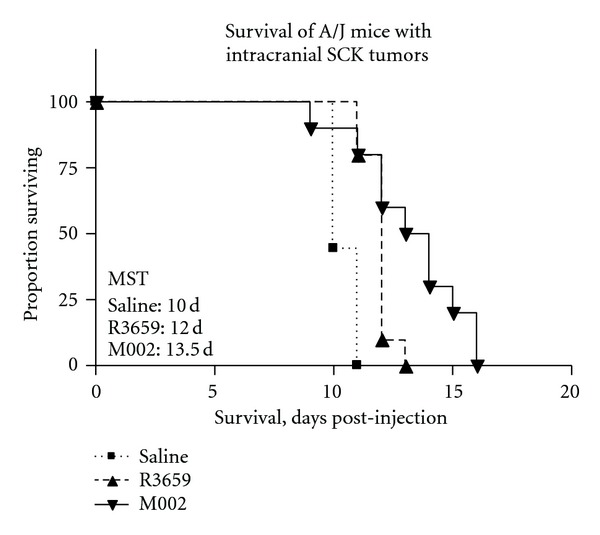
Kaplan-Meier plots of antitumor effect of M002 *in vivo* against intracranial SCK tumors. Tumors were treated four days after establishment with 1.5 × 10^7^ PFU of R3659 or M002, or were given saline only as a control. By log-rank analysis, both R3659-(*P* = 0.0004) and M002-treated mice (*P* = 0.0012) survived significantly longer than mock-treated mice, and M002-treated mice survived longer than R3659-treated mice (*P* = 0.0333). Median survival times (MSTs) are indicated.

Having established an immunocompetent, intracranial model of breast cancer brain metastasis, we then employed this model in an evaluation of M002 efficacy *in vivo*. Intracranial tumors of SCK cells were established in A/J mice as previously, although a lower dose was used (2.5 × 10^3^). After four days, tumors were injected with 1.5 × 10^7^ PFU of either M002 (*n* = 10) or R3659 (*n* = 10), or with sterile saline only (*n* = 9) as a control. The mice were then monitored for neurological symptoms, at which point they were euthanized. Mice given saline only exhibited a median survival of 10 days (Figure 6). By log-rank analysis, both R3659- and M002-treated treated mice survived significantly longer than mock-treated mice, with median survival times of 12 days (R3659, *P* = 0.0004) and 13.5 (M002, *P* = 0.0012) days. Additionally, the M002-treated mice survived longer than the R3659-treated mice (*P* = 0.0333). Two mice treated with M002 survived for 16 days, or 60% longer than the median survival exhibited by the mock-treated mice. These results demonstrate in a proof-of-principle experiment that M002 treatment can prolong survival in an immunocompetent model of breast cancer brain metastases.

## 4. Discussion

 In this paper, we present proof-of-principle data supporting the use of an IL-12-expressing oncolytic HSV as a novel treatment for breast cancer brain metastases. We have demonstrated a number of key points. First, we have confirmed in both human breast cancer and murine mammary carcinoma cells that infection with M002 produces IL-12 at physiological levels. Next, we have shown that a variety of breast cancer cell lines support the robust replication of *γ*_1_34.5-deleted viruses such as M002, although the replication is more limited in the murine tumor cells examined. We have also demonstrated that these viruses mediate a direct cytotoxic effect on breast cancer cells. Finally, we have described an immunocompetent model of breast cancer brain metastases and shown that M002 improves the survival of treated animals to a greater extent than does a replicating, non-IL-12-expressing control virus in this model. 

 We observed robust IL-12 production, as measured by ELISA, in all three of the cell lines assayed. The levels obtained from the murine cells (SCK, 4T1) were, in general, 10-fold higher than those obtained from the human MDA-MB-361 cells. We speculate that this may have been the result of cell death in the human cells, as this line was more susceptible to M002 replication than were the murine lines. Rapid oncolysis of the MDA-MB-361 cells may therefore have precluded high levels of IL-12 production. Alternatively, the EGR-1 promoter used to drive expression of the IL-12 transgene may have higher activity in the murine lines versus the human line, as our informal observations suggest that the SCK and 4T1 lines proliferate more rapidly than does the MDA-MB-361 line. Nonetheless, we have confirmed that breast cancer cells support IL-12 production from M002. The amount of IL-12 that we observed was similar to those in previously published studies of M002 in neuroblastoma [25] and glioma [28] cell lines.

 We have also shown robust viral replication of the *γ*_1_34.5-deleted virus M002 in a panel of human breast cancer cell lines. This panel was chosen to represent some of the key risk factors associated with brain metastases, such as younger age, invasive ductal carcinoma type, HER2 overexpression, and “triple negative” receptor status. Moreover, with the exception of normal like, each of the major molecular breast cancer subtypes was represented in these studies. Although the results obtained in an individual cell line may not necessarily be reflective of an entire subtype, we did not observe any correlation between replication efficiency and subtype, suggesting that M002 may be effective against a range of breast cancer types, and that brain metastases are not likely to be resistant to oncolytic HSV replication. It is of note that in each of the single-step replication experiments that were performed, the MDA-MB-231 line consistently yielded among the highest titers, suggesting that basal-like/triple-negative breast cancers, which are particularly resistant to established therapies, are relatively permissive for oncolytic HSV replication. 

In the course of these studies, we have developed a reliable, reproducible, and facile model of breast cancer brain metastasis. The establishment of brain tumors by direct injection of SCK cells into the brains of mice has, to our knowledge, not been previously reported. The seeding of brain metastases from a primary tumor remains an ideal model, as this would be more physiologically relevant with respect to tumor distribution and would recapitulate all of the steps of the metastatic cascade. However, practical considerations limited the feasibility of this approach. SCK tumors are reported to be metastatic *in vivo*, but their propensity for establishing brain metastases has not been characterized. Also, given the current limitations of systemic delivery of oncolytic viruses, direct intracranial implantation of tumor cells remains a facile and reliable approach to generate tumors at known locations in the brain for evaluating oncolytic viruses *in situ*. Moreover, this approach is independent of the potentially confounding variables involved with intravascular virus delivery, such as complement binding, neutralizing antibody response, or nonspecific sequestration of virus in nontarget tissues. Nonetheless, breast cancer brain metastases typically present as a discrete number of lesions (1–5) with well-circumscribed borders, which are accessible surgically [48] or provide unambiguous stereotactic radiosurgery targets [49], and therefore the potential for direct intratumoral inoculation in the clinical setting exists.

 In a treatment experiment, we have shown that a single injection of M002 extended the survival of treated mice over that of mock-treated mice, and did so more effectively than a non-cytokine control virus. This finding is in agreement with our earlier studies of primary brain tumors and is particularly noteworthy considering the aggressiveness of the model. Although treatment with M002 extended the survival of the mice, the single dose used here was not sufficient to yield tumor eradication. Histological examination of the treated mice confirmed that continued tumor growth was the cause of death in all three treatment groups. The SCK model described here has certain advantages for the evaluation of M002, namely, permissiveness for HSV replication and the establishment of well-circumscribed brain tumors *in vivo*. However, the aggressiveness of this model and the rapid onset of morbidity in tumor-bearing mice are key disadvantages. We speculate that the short time frame of this model prevented an even greater antitumor effect being mediated by M002, since prior studies indicate that treatment with M002 stimulates an influx of immune cells including CD4+ and CD8+ T cells, macrophages, and NK cells that peaks 6-7 days post-injection of virus [25, 28], which is near the median survival observed in this model. It is possible that earlier treatment or lower input dose of cells would have allowed for a greater antitumor effect. Although we have shown in this proof-of-principle experiment that M002 improves survival more effectively than a non-IL-12 oncolytic HSV, additional testing to elucidate which of the various antitumor mechanisms (i.e., viral oncolysis, immune cell infiltration, or antiangiogenesis) were chiefly responsible for the improved survival in this model would be valuable.

The potential utility of oncolytic viruses against breast cancer has been established using a number of viral systems, including HSV [50, 51]. However, only a limited number of studies have been published regarding the use of oncolytic HSVs against breast cancer brain metastases [23, 52, 53]. These include one study in which an IL-12-expressing mutant HSV did not improve survival in an immunocompetent model over that of a control virus [52], although differences in either the viral or model system employed may explain the discrepancy between this study and our findings. 

## 5. Conclusions

The clinical problem of brain metastases from breast cancer is one of ever-increasing significance, and effective therapies for these lesions are urgently needed. Here, we provide rationale for further studies of an IL-12-expressing *γ*_1_34.5-deleted HSV as a treatment for breast cancer brain metastases. We have demonstrated that an IL-12-expressing *γ*_1_34.5-deleted HSV efficiently replicates in a panel of breast cancer cells representative of brain metastases and that infection of breast cancer cells yields physiologically relevant levels of IL-12. Treatment of intracranial tumors with this virus in an immunocompetent model of breast cancer brain metastasis improves survival more effectively than treatment with a non-cytokine control virus. We therefore conclude that an IL-12-expressing *γ*_1_34.5-deleted virus may be an effective new therapy for this disease, and further testing is warranted. While this study was, for practical reasons, conducted with an HSV expressing murine IL-12, a similar virus expressing human IL-12 has already been prepared by our research group, and a clinical trial for this virus in glioblastoma patients is planned. Therefore, the possibility exists that this virus could also be made available for a breast cancer trial.

## Figures and Tables

**Table 1:** Key features of the human breast cancer cell lines used in this study. Some lines can be classified into more than one subtype. Expression of ER and PR and amplification/overexpression of HER2 are indicated (+).

Cell line	Cancer type	Molecular subtype	Age of donor	Site of isolation	Receptor status			References
					ER	PR	HER2	
BT-474	IDC	Luminal A/B	60	Primary tumor	−	+	+	[11–14]
MDA-MB-361	AC	Luminal A	40	Brain	+	+	+	[12, 13, 15–17]
MDA-MB-231	AC	Basal	51	Pleural effusion	−	−	−	[13–17]
SK-BR-3	AC	Luminal B/HER2	43	Pleural effusion	−	−	+	[12–14, 18]
ZR-75-1	DC	Luminal A	63	Ascites	+	+	+	[12–14, 19]

AC: adenocarcinoma, DC: ductal carcinoma, ER: estrogen receptor, HER2: human epidermal growth factor receptor 2, IDC: invasive ductal carcinoma, and PR: progesterone receptor.
